# Ischemia-Reperfusion Injury and Volatile Anesthetics

**DOI:** 10.1155/2014/526301

**Published:** 2014-01-02

**Authors:** Engin Erturk

**Affiliations:** Department of Anesthesiology and Intensive Care Medicine, Faculty of Medicine, Karadeniz Technical University, 61080 Trabzon, Turkey

## Abstract

Ischemia-reperfusion injury (IRI) is induced as a result of reentry of the blood and oxygen to ischemic tissue. Antioxidant and some other drugs have protective effect on IRI. In many surgeries and clinical conditions IRI is counteract inevitable. Some anesthetic agents may have a protective role in this procedure. It is known that inhalational anesthetics possess protective effects against IRI. In this review the mechanism of preventive effects of volatile anesthetics and different ischemia-reperfusion models are discussed.

## 1. Introduction

After the ischemic period reentry of the blood to tissue causes massive release of oxygen free radicals. These free radicals trigger enzymatic reactions, such as peroxidation of polyunsaturated fatty acids or plasma lipoproteins, which leads to oxidative destruction of cell membranes and the productions of toxic reactive metabolites and cell injury involving DNA, proteins, and lipids [1]. All of these events are called ischemia-reperfusion injury (IRI).

## 2. Pathophysiology and Clinical Presentation

IRI occurred mostly during anesthesia and intensive care practice. In cardiac surgery or tourniquet application for extremity surgery, thromboembolic events and revascularization, severe hypotension, and restoration of hypovolemic shock, in organ transplantation, can cause IRI. During the ischemia anaerobic glycolysis is activated and then establishment of reperfusion accompanied by pro- and anti-inflammatory cytokine release, polymorphonuclear neutrophil activation, and platelet adhesion to the vascular endothelium occur with production of reactive oxygen species and release of vasoactive factors [2–4]. On the other hand, plasma concentration of some enzymes such as catalase, glutathione peroxidase, superoxide dismutase, lactated dehydrogenase, and some metabolites such as malonyldialdehyde (MDA), ischemia-modified albumin (IMA), lactate, and reactive oxygen species (ROS) increases during postreperfusion period. As a result of these pathophysiological phenomena, local and systemic inflammatory responses are formed by different mechanisms [5–7].

The total antioxidant status (TAS) of human body counteracts oxidative stress and reperfusion injury. It was found that while ROS increased, TAS decreased as a result of oxidative stress [8]. However, most patients do not counteract severe complication despite increasing ROS. It was explained that patients with normal TAS can tolerate these negative effects of oxidative stress. However, advanced age, severe ill, traumatic, or cancer patients have lower TAS in their plasma [9, 10]. In these patients oxidative stress may cause destruction of DNA and some structures with protein and lipid.

Severe systemic inflammatory reactions as a result of massive inflammatory mediator release and reperfusion injury may activate endothelial cells in remote organs which are not exposed to initial ischemic injury [11]. The distant effect of ischemia reperfusion causes microvascular injury with leukocyte invasion on endothelium [12]. These events may lead to multiorgan failure and increased postoperative morbidity and mortality. It was reported that IRI may cause cardiopulmonary complication such as tachyarrhythmia and hypoxia [13].

A lot of studies are conducted to prevent IRI. Some of these are related to anesthesia method such as regional anesthesia, inhalation general anesthesia, or total intravenous anesthesia.

## 3. The Mechanisms of Protective Effects of Volatile Anesthetics

The effects of volatile anesthetics on IRI were investigated for several years [14–17]. It is known that volatile anesthetics, especially halogenated, have a protective role against IRI. These protective effects have been attributed to pre- and postconditioning effects with apoptosis. The mechanisms of these effects have been investigated, and new pathways are asserted continuously. Kowalski et al. [18] stated that polymorphonuclear neutrophils (PMN) lead to reperfusion injury in many organs and tissues via adhesioning to vascular endothelial cells. They investigated the effects of halothane, isoflurane, and sevoflurane on postischemic adhesion of human PMN in the intact coronary system of isolated perfused guinea pig hearts. As a result of this study they found that volatile anesthetics had inhibitory effect on ischemia induced adhesion of PMN and concluded that it may be beneficial for the heart during general anesthesia. Similarly, it was stated that volatile anesthetics were able to modulate the interaction of PMN with the endothelial cell, and this may play a crucial role in the initiation of IRI in other studies [17, 19].

However, protective effects of volatile anesthetics against IRI are wondered and some studies were carried out to explain the mechanism. Novalija et al. [20] performed anesthetic preconditioning with sevoflurane and gained positive outcomes with isolated guinea pig hearts. They explained the positive effect of sevoflurane with improved adenosine triphosphate synthesis and reduced ROS formation in mitochondria after ischemia by a redox dependent mechanism. Kersten et al. [21] stated that volatile anesthetics improved recovery of contractile function of postischemic, reperfused myocardium, and activated KATP channels. For the same purpose Zaugg et al. [22] studied to test whether volatile anesthetics mediate this effect by activation of the mitochondrial adenosine triphosphate-sensitive potassium (mitoKATP) or sarcolemmal KATP channel in rat ventricular myocytes and to evaluate the signaling pathways involved. At the end of their study they found that volatile anesthetics mediate their protection in cardiomyocytes by selectively priming mitoKATP channels through multiple triggering protein kinase C-coupled signaling pathways. And also in another study Marinovic et al. [23] investigated “an innative” protective mechanism of volatile anesthetics against IRI. They made an effort to reveal whether KATP channels are triggers initiating the preconditioning signaling and/or effectors responsible for the cardioprotective memory and activation during ischemia reperfusion. Adult rat cardiomyocytes were exposed to oxidative stress. To induce preconditioning, the myocytes were pretreated with isoflurane. The involvement of sarcolemmal and mitochondrial KATP channels was investigated using specific inhibitors. At the end of the study they concluded that both sarcolemmal and mitochondrial KATP channels play essential and distinct roles in protection afforded by isoflurane. They also stated that sarcolemmal KATP channel seemed to act as an effector of preconditioning, whereas mitochondrial KATP channel played a dual role as a trigger and an effector.

Lucchinetti et al. [24] explored in their study the effects of sevoflurane, propofol, and intralipid on metabolic flux rates of fatty acid oxidation (FOX) and glucose oxidation (GOX) in hearts exposed to ischemia and reperfusion. They studied on isolated, paced working rat hearts that were exposed to 20 min of ischemia and 30 min of reperfusion. Study groups were treated with sevoflurane or propofol. They observed that sevoflurane improved the recovery of left ventricular work and myocardial efficiency. This functional improvement was accompanied by reduced increases in postischemic diastolic and systolic intracellular Ca^+2^ concentrations. Sevoflurane increased GOX and decreased FOX in hearts exposed to ischemia and reperfusion. GLUT4 expression was markedly increased in lipid rafts of sevoflurane treated hearts. Increased GOX closely correlated with reduced Ca^+2^ overload. As a result of their study they concluded that enhanced glucose uptake via GLUT4 fuels recovery from Ca^+2^ overload after ischemia and reperfusion in sevoflurane treated hearts.

The protective effects of volatile anesthetics have been longstanding subject in many studies. It was shown that one of the mechanisms of IRI was an intracellular calcium overload. Volatile anesthetics might also protect the myocardium from IRI by altering myocardial calcium fluxes. They also preserve myocardial energetics and protect from ROS derived injury. Louvier and Lançon stated that enflurane and halothane seemed to be more efficient than isoflurane. They explained these cardiovascular effects by a specific effect on myocardial cells. They also stated that halothane and enflurane mainly decreased intracellular calcium availability by a direct effect on sarcoplasmic reticulum, while isoflurane only decreased the transsarcolemnal calcium entry [25].

## 4. Organ Specific IRI Models and Volatile Anesthetics

Oxidative stress and reperfusion injury may develop in different ischemia-reperfusion models. In these models enzymatic reactions and cellular destructions can affect not only related system but also remote organ and system. Multiorgan involvement may occurr as a result of IRI. The main organs in which IRI occurs are heart, lung, brain, liver, kidney, and intestine. Preconditioning and postconditioning with volatile anesthetics confer protection against reperfusion injury in these organs. There are a lot of studies that investigated protective effects of volatile anesthetics on these organs.

### 4.1. Heart Surgery and IRI

One of the most studied reperfusion model is open heart surgery. It was known that cardiac surgery using cardiopulmonary bypass is associated with release of inflammatory mediators and severe systemic inflammatory reactions. Cardiopulmonary bypass was performed and heart was exposed to ischemia in this surgery. After declamping the aorta ischemic tissue was reperfused and reoxygenated. Reperfusion and reoxygenation of the myocardium may lead to dysrhythmia or hypotension called “myocardial stunning.” That is why the effects of volatile anesthetics on reperfusion injury were mostly studied on open heart surgery. On the other hand some antioxidative agents were used for prevention of IRI in both clinical and experimental studies. Propofol is chemically similar to phenol based on free radical scavengers and endogenous antioxidant vitamin E [26, 27]. Therefore, it was used in many clinical and experimental reperfusion injury studies. In consequence of these studies, it was usually concluded that propofol shortens and attenuates oxidative stress and IRI [28–32]. As the protective effects of propofol is well known, some studies compared propofol and volatile anesthetics such as halothane, isoflurane, and sevoflurane carried out to exhibit the effect of volatile anesthetics on oxidative stress and IRI. Conzen et al. [33] carried out a study on 20 patients scheduled to undergo elective offpump coronary artery bypass surgery. They maintained anesthesia with either sevoflurane or propofol. For assessing myocardial injury, troponin I and myocardial fraction of creatine kinase were determined during the postoperative 24 hours. They found that troponin I concentration increased significantly in propofol infusion group. They concluded that cardiac output improved with sevoflurane but not with propofol, suggesting better maintenance of myocardial function. In another study Julier et al. [34] investigated the effects of sevoflurane preconditioning on myocardial and renal function by measuring postoperative release of brain natriuretic peptide. They found that sevoflurane preconditioning significantly decreased postoperative release of brain natriuretic peptide and concluded that sevoflurane preconditioning preserves myocardial and renal function. Garcia et al. [35] stated at the end of their study that pharmacological preconditioning by sevoflurane provided protective role in cardiac events in coronary bypass patients.

### 4.2. Thoracic Surgery/One Lung Ventilation and IRI

One lung ventilation (OLV) is frequently used for thoracic and some other surgeries. During OLV, vessels in nonventilated lung (NVL) are constructed and blood flow mainly goes towards other lung lob. In such condition called hypoxic pulmonary vasoconstriction (HPV), while the blood flow of other lobe increases, perfusion and oxygenation of NVL decrease. As a result of this, tissue ischemia occurs in nonventilated site. After resuming 2-lung ventilation, reentry of the blood to ischemic tissue causes sudden and significant increase in ROS production leading to IRI.

We carried out a study to compare the preventive effects of sevoflurane and propofol from IRI in thoracic surgery with OLV by measuring blood gases, IMA, and MDA. We observed lower arterial oxygen pressure in sevoflurane group than in propofol group during OLV as a demonstration of HPV. IMA level at postoperative sixth hour was lower in sevoflurane group than in propofol group. We conclude that sevoflurane was superior in preventing in IRI compared to propofol [36]. Casanova et al. [14] emphasized importance of ischemia-reperfusion induced lung injury in thoracic surgery due to association with ventilation damage to one lung. They studied and evaluated the cytoprotective effects of sevoflurane compared with propofol in a pulmonary autotransplant model in pigs. They found increased oxidative stress markers and proinflammatory mediators in the propofol group. In a consequence of their study, they concluded that sevoflurane decreased the inflammatory response and oxidative stress and provided a protection in a live ischemia reperfusion lung model. In another study Liu et al. [37] investigated the effects of administration of isoflurane and sevoflurane before the intervention on ischemia-reperfusion induced lung injury in an isolated buffer-perfused rat lung model by measuring the coefficient of filtration of the lung, lactate dehydrogenase activity, tumor necrosis factor alpha, nitric oxide metabolites in the perfusate, and the wet-to-dry lung weight ratio. They found that administration of 1 MAC isoflurane or sevoflurane before ischemia significantly attenuates ischemia-reperfusion induced filtration and the wet-to-dry lung weight ratio. This 1 MAC inhibits increase of lactate dehydrogenase activity and tumor necrosis factor alpha in the perfusate and abrogates the decrease in nitric oxide metabolites in the perfusate. They concluded that isoflurane and sevoflurane administered before ischemia could attenuate ischemia-reperfusion induced injury in isolated rat lungs.

### 4.3. Tourniquet Induced Extremity IRI

Another frequently studied model is tourniquet induced ischemia-reperfusion model. Application of tourniquet is liberally used for providing bloodless surgical field and control of intraoperative bleeding in extremity surgery. Therefore, muscle ischemia occurs in distal area of tourniquet. IRI occurs after deflation of tourniquet and reoxygenation of ischemic tissue. It was stated that muscle ischemia is accompanied by hypoxic cellular challenge and anaerobic glycolysis, reperfusion by neutrophil activation, formation of reactive oxygen species, and release of vasoactive factors [2]. Carles et al. [38] investigated the effects of sevoflurane compared with propofol in tourniquet induced ischemia reperfusion by measuring with microdialysis probes interstitial metabolite levels of anaerobic glycolysis. They found that lactate, pyruvate, and glucose remained at a significantly higher level in the sevoflurane group during reperfusion. Their results indicated that there is a better availability of interstitial glycolysis metabolites in the skeletal muscle during ischemia and reperfusion after sevoflurane exposure. They concluded that sevoflurane had a potential preconditioning effect on tourniquet-induced skeletal muscle IRI. It may be considered that there is a more efficient anaerobic glycolysis after sevoflurane exposure because of higher availability of energetic substratum, that is, pyruvate, allowing higher production of lactate and therefore higher mitochondrial ATP [39, 40]. Higher interstitial glycolysis substratum levels resulting from sevoflurane exposure may participate in the preservation of ATP synthesis in the skeletal muscle.

### 4.4. Major Vascular Surgery and IRI

In some surgical procedures, such as aneurysm repair of big vessels, traumatic vessel injuries, and procedures related to artery being clamped to provide bloodless surgical area, volatile anesthetics were used to show their preventive effects from IRI in these procedures. Aortic ischemia and reperfusion may induce pulmonary sequestration of neutrophil granulocytes. Kalb et al. [41] investigated the effects of pre- or postconditioning with sevoflurane showing pulmonary neutrophil accumulation after IRI of the aorta. Anesthetized and mechanically ventilated rats underwent laparotomy and developed ischemia by clamping of the infrarenal aorta. Pre- and postconditioning with sevoflurane were applied. Following reperfusion, the lungs were removed for microscopic determination of neutrophil accumulation. They found that preconditioning, but not postconditioning, with sevoflurane reduced pulmonary neutrophil accumulation after IRI. They concluded that since neutrophil accumulation played a major role in the pathophysiology of acute lung injury, their data suggested a protective effect of sevoflurane preconditioning on remote pulmonary IRI.

Annecke et al. [15] compared the effects of sevoflurane with propofol on IRI after thoracic aortic occlusion in pigs. The animals received sevoflurane or propofol anesthesia before, during, and after lower body ischemia. Fluid and catecholamine requirements were assessed. Serum samples and intestinal tissue specimens were obtained. All animals displayed a severe reperfusion injury following 90 min occlusion of the thoracic aorta. However, animals receiving sevoflurane showed less signs of IRI as assessed by systemic hemodynamic instability than animals receiving propofol for the same intervention. Norepinephrine requirement in the sevoflurane group was significantly reduced during reperfusion. Animals tested with sevoflurane had a less pronounced increase of serum enzyme activities indicative of tissue injury. Serum activities of lactate dehydrogenase, aspartate transaminase, and alanine aminotransferase were lower with sevoflurane. In a consequence of the study they concluded that use of sevoflurane compared with propofol attenuated the hemodynamic sequelae of IRI. Koşucu et al. [42] investigated the effects of sevoflurane anesthesia combined with epidural anesthesia on IRI in patients undergoing surgical revascularization due to aortoiliac occlusive disease, by measuring plasma MDA and IMA levels. They found that serum levels of MDA and IMA were lower in study group compared to control group. In consequence of their study, they concluded that the sevoflurane anesthesia combined with epidural anesthesia might decrease the IRI in aortoiliac occlusive disease.

### 4.5. Cerebral Events and IRI

Cerebral IRI is encountered from various neurological, vascular, and cardiovascular procedures. This typically causes a disorder of water homeostasis and has been associated with oxidative stress, inflammatory response, lipid peroxidation, and apoptosis [43–46]. Preconditioning with volatile anesthetics can limit the cerebral IRI. Bedirli et al. [47] carried out a study to examine the effects of sevoflurane or isoflurane preconditioning on cerebral ischemia-reperfusion induced inflammation, oxidative stress, and lipid peroxidation and test the hypothesis that the underlining mechanism of the protective effect of preconditioning involves changes in the apoptotic gene expression profiles in an experimental model of middle cerebral artery occlusion in rats. In consequence of their study they found that sevoflurane and isoflurane preconditioning ameliorates inflammation, cerebral lipid peroxidation, and histologic injury. They also concluded that downregulation of proapoptotic molecules and upregulation of antiapoptotic molecules may be associated with this effect.

For the same purpose Wang et al. [48] investigated the postconditioning neuroprotective effect of sevoflurane in rats with middle cerebral artery occlusion. They found that postconditioning with sevoflurane not only reduced infarct volume but also improved learning and memory. They concluded that this neuroprotective effect may be partly due to the activation of PI3K/Akt pathway and inhibiting neuronal apoptosis. In another study, Ishiyama et al. [49] compared the effects of sevoflurane with propofol on cerebral pial arteriolar and venular diameters during global brain ischemia and reperfusion. Twenty rabbits were anesthetized with sevoflurane or propofol and then global brain ischemia was induced by clamping the brachiocephalic, left common carotid, and left subclavian arteries. They observed pial microcirculation microscopically through closed cranial windows and measured using a digital video analyzer. They found that pial arterioles and venules did not dilate immediately after reperfusion and subsequently constricted throughout the reperfusion period in propofol group. In contrast, pial arterioles and venules dilated temporarily and returned to baseline in sevoflurane group. Adverse effects in sevoflurane group (pulmonary edema and acute brain swelling) were higher than propofol group. In addition, blood pressure, heart rate, and plasma glucose were stable in sevoflurane group.

### 4.6. Liver and IRI

Temporary interruption of blood flow of liver causes hepatic ischemia. IRI may cause removal of interruption and subsequent reperfusion in some surgical procedures. Since volatile anesthetics are capable of providing relevant organ protection from IRI, several studies are conducted in this field [50, 51]. Bedirli et al. [52] investigated the effects of isoflurane and sevoflurane in a warm liver ischemia-reperfusion model on cytokines, hepatic tissue blood flow, energy content, and liver structure. They found that sevoflurane given before, during, and after hepatic ischemia protected the liver against IRI, whereas the effects of isoflurane on hepatic IRI were not notable.

### 4.7. Kidney and IRI

Renal IRI is an inflammatory process involving multiple cellular and systemic responses, including complement activation, activation of proinflammatory cytokines and chemokines, and infiltration by leukocytes such as neutrophils, macrophages, and T cells [53]. Lee et al. [54] reported that volatile anesthetics, including isoflurane, protected from renal IRI injury by attenuating the inflammatory response as well as necrosis. Daqing et al. [55] used preconditioning with noble gas, xenon, to show its protective effect in renal IRI. They observed that xenon was a natural inducer hypoxia-inducible factor 1a. Providing their data confirmation in the clinical setting, they suggested that xenon preconditioning before renal ischemia can prevent acute renal failure arising from IRI. Guye et al. [56] examined a possible protective effect of desflurane preconditioning on the kidney in renal ischemia-reperfusion model in rabbits. They investigated tubular cell damage histologically. They found lower histological damage in desflurane group and concluded that desflurane preconditioning reduced renal IRI.

### 4.8. Opposing Views

There are lots of studies in the literature investigating the effects of volatile anesthetics in other ischemia-reperfusion models. However, recently, many of these studies have been performed with sevoflurane and isoflurane. Halothane and enflurane are no longer investigated for this purpose due to decreased usage of these agents. However, some studies suggested that there is no protection [29, 57, 58], moreover harmful [59] effects of volatile anesthetics.

### 4.9. In the Future

IRI may occur in different clinical conditions without surgical intervention. After successful cardiopulmonary resuscitation, reperfusion is established in tissues and different organs that remained ischemic and hypoxic. As a result of reperfusion, IRI is inevitably encountered. On the other hand, severe hypotension connected with hypovolemic, hemorrhagic, or septic shocks also causes tissue hypoxemia. After treatment of these clinical conditions IRI may also occur.

Recently, tissue and organ transplantation is rapidly improved. As the transplanted organs are exposed to ischemia and reperfusion, IRI will be encountered more frequently. Therefore novel treatment modality will be offered to clinical practice. Perhaps usage of volatile anesthetics may be a part of this in the future.

## 5. Conclusion

Although there are a lot of studies in the literature suggesting potential protective effects of volatile anesthetics, further studies are required to show the effects of volatile anesthetics on IRI.
